# Reviewed article: Muñoz, FL; Cruz, RF; Silveira, LVA, Diaz, MP. Latent dimension of comorbidities and depression in older adults: a cross-sectional study using National Health Survey data, Brazil, 2019. Epidemol Serv Saude. 2026:35;e20250722

**DOI:** 10.1590/S2237-96222026v35e20250722.b

**Published:** 2026-10-12

**Authors:** Cristine Vieira do Bonfim

**Affiliations:** 1 Fundação Joaquim Nabuco, Recife, PE, Brazil

## First round comments

O Núcleo Editorial da revista Epidemiologia e Serviços de Saúde (RESS) informa que a revisão do manuscrito intitulado “A Dimensão Latente de Comorbidade Está Associada à Ocorrência de Depressão em Idosos Brasileiros: Achados da Pesquisa Nacional de Saúde de 2019, utilizando o Modelo de Equações Estruturais Generalizadas” (RESS-2025-0722) foi concluída. O manuscrito foi considerado com potencial para publicação, mas reformulações maiores são necessárias. Solicitamos que apreciem a avaliação e realizem a revisão do manuscrito.

Para fazer a revisão do manuscrito, efetuem login no sistema e acesse o Author Center, onde o encontrará na lista “Manuscripts with Decisions”. Abaixo da barra “Actions”, clique em “Create a Revision”.

Lembrem-se de iluminar as mudanças realizadas no documento colorindo o texto manualmente com o amarelo. Uma vez terminada a revisão, o manuscrito deve ser submetido por meio do Author’s Center.

Todos os comentários do Núcleo Editorial e dos(as) revisores(as) devem ser considerados e respondidos, item por item, em carta aos editores. Para agilizar o processamento do manuscrito revisado, pedimos os comentários sejam bem específicos (informando página e linha). As sugestões que não forem consideradas deverão ser justificadas com fundamentação científica.

Solicita-se, gentilmente, que não deixem para enviar o manuscrito no último dia, pois o sistema não é nacional e sempre irá expirar o prazo no dia limite.

Agradecemos novamente pela submissão do artigo à RESS.

Cordialmente,

Núcleo Editorial

Epidemiologia e Serviços de Saúde: revista do Sistema Único de Saúde do Brasil

Site: www.scielo.br/ress

Comentários maiores

O manuscrito necessita de revisão linguística, existem frase truncadas. Termos que são característicos de IA. Uso do plural majestático.

Título

Ajustar o título, conforme exemplo: “Tema principal: tipo de delineamento, local, ano-ano”. Solicita-se que o título seja mais sucinto, favorecendo a sua compreensão. Observa-se a presença de elementos metodológicos que não favorecem a compreensão. Sugere-se adotar o modelo indicado pela RESS.

Resumo

Sugere-se que a redação seja feita como frases curtas e diretas para facilitar o entendimento.

Objetivo - Iniciar diretamente com o objetivo (identificar ...). Acrescente-se que o objetivo apresentado ao final da introdução deve ser igual ao do resumo (copiar e colar).

Métodos

O número de participantes deve ser deslocado para a seção de resultados. Consiste na primeira informação a ser relatada.

A informação sobre a variável latente deve anteceder a análise dos dados.

A forma de descrição da análise está extensa, com muitas repetições do termo modelo. Por favor, tornem mais direta e acessível.

Resultados

Iniciar com o número total de participantes.

Inserir dados numéricos para apoiar as afirmações. Trata-se da seção de resultados de um estudo epidemiológicos, os dados numéricos são necessários e relevantes.

Descrever os resultados numéricos não apenas como derivados, por exemplo, as percentagens, mas também como números absolutos dos quais os derivados foram calculados e especificar a significância estatística a eles relacionada, se pertinente.

O dado apresentado no texto sobre a prevalência de depressão é de 11,8%, que está diferente no resumo. Lembrar que deve ser relatado o dado exato, sem arredondamentos. Acrescente-se que essa taxa não foi localizada nas tabelas.

A definição da variável latente “comorbidades” deve ser feita na seção de métodos.

Conclusão

Deve ser concisa e deter-se ao que se pode afirmar a partir dos resultados apresentados no resumo em resposta ao objetivo.

Ajustar o resumo, conforme as sugestões. O número máximo de palavras é 250.

Palavras-chave

Incluir descritor referente ao desenho do estudo como último termo.

Aspectos éticos

Citar a Resolução 674 de 2022.

Introdução

A seção tem a finalidade de demonstrar a relevância do problema estudado para a saúde pública, incluir dados epidemiológicos da magnitude do problema estudado, com citações de referências atuais. Observa-se na seção a presença de várias frases sem as citações que as fundamentam. Acrescente-se a presença de referências que necessitam de atualização. A RESS sugere que sejam utilizadas referências de artigos científico, publicados em revistas indexadas nos últimos cinco anos.

Página 2, linhas 6-8, - Informar onde e quando, além de incluir os dados epidemiológicos. Essa frase faz parecer que somente o aumento das pessoas idosas, contribui para o aumento da depressão nesse segmento populacional, desconsiderando outros fatores relacionados com a depressão nessa população.

Página 2, linha 10 - Incluir os dados que mostram esse crescimento.

Página 2, linha 16 - especificar qual é o marco demográfico.

Incluir a justificativa para o estudo, descrevendo a sua contribuição, relevância e inovação. A justificativa deve anteceder o objetivo. Lembrar que o objetivo do resumo e da introdução devem ser iguais. Parece que o texto apresentado, justifica apenas a forma de analisar os dados.

O objetivo deve informar o quê foi investigado, onde e quando. A versão atual do objetivo precisa ser mais específica sobre o conteúdo da investigação.

Métodos

Recomenda-se aderir consistentemente ao checklist STROBE para relato de estudos observacionais. A seção de métodos deve ser ajustada e as informações devem ser desmembradas e completadas de acordo com os seguintes itens: Delineamento do estudo; Contexto; Participantes; Variáveis (inserir todas as variáveis e categorias investigadas de forma idêntica ao que consta nos resultados e ilustrações); Fontes de dados/ mensuração; Viés; Tamanho do estudo; Métodos estatísticos. Acesse: https://www.strobe-statement.org/

O número de participantes deve ser apresentado somente na seção de resultados.

Para colesterol alto incluir o ponto de corte utilizado.

Para o número de moradores incluir a categorização.

As categorias das variáveis devem ser iguais aquelas descritas nas tabelas. Escolaridade está diferente. A variável número de moradores está com o nome de co-residentes. Importante revisar para que ocorra correspondência entre os métodos e resultados.

Incluir referência para Modelos de Equações Estruturais Generalizadas.

Sugere-se substituir idade por faixa etária.

Na página 4, linha 24 - a varável “Escolaridade” conforme a tabela 1, aparece com o nome de “nível de escolaridade”. Revisem com bastante atenção variáveis na seção de métodos e nos resultados.

Resultados

Iniciar com o número total de participantes do estudo.

Apresentar sempre as frequências absolutas e as relativas.

A descrição dos resultados deve seguir a sequência das variáveis citadas na tabela.

A variável sexo adotou a categorização em Feminino e Masculino, é essa forma que deve ser preservada no texto.

Página 5, primeiro parágrafo - qual a tabela que apresenta o resultado do modelo de mensuração? Citar a tabela no texto. Lembrando que os dados citados no texto, precisam ser contemplados nas ilustrações (tabelas e figuras).

Página 5, segundo parágrafo - cita resultados não apresentados em tabela. Por favor, incluam os resultados em tabelas.

Novamente chama-se a atenção para a nomenclatura utilizada. Na seção de métodos as categorias das variáveis apresentam um nome e na seção de resultados outros. O texto utiliza um nome e a Figura outro. Por exemplo: a Figura 2, utiliza feminino e masculino e o texto descreve homens e mulheres. Por favor, padronizem em feminino e masculino.

Página 5, linhas 33-35 - parece interpretação dos resultados. Ressalte-se que os resultados devem ser descritos e não interpretados nessa seção.

Página 6, primeiro parágrafo - Qual a tabela que apresenta esses resultados?

Discussão

De forma geral, a discussão precisa estabelecer a conexão entre os principais achados do estudo com a literatura, respeitando a mesma sequência da seção de resultados. Esse um apontamento que precisa ser bastante trabalhado, uma vez que não fica evidente no texto qual o resultado que está sendo discutido.

Apresentar breve (primeiro parágrafo) resumo dos principais achados, com ênfase na forma como estes contribuem para aumentar o volume de conhecimento sobre o assunto. Na versão atual o primeiro parágrafo traz uma revisão de literatura, sem conexão com o resultado.

Apresentar as limitações do estudo, levando em consideração fontes potenciais de viés ou imprecisão no segundo parágrafo e os métodos usados para minimizar as limitações. Na versão atual esse parágrafo apenas descreve o que foi feito, do ponto de vista metodológico.

Após esclarecer as limitações da pesquisa, o texto da discussão deve ser guiado pelos seus achados. Interpretar cada resultado relevante e discuti-lo à luz da literatura. Esgotar o tema abordado e evitar retomá-lo adiante no texto de modo circular e redundante. Evitar parágrafos sem conexão com a literatura ou que somente a revisam, sem discussão com os resultados. Assegurar que a discussão faz a ponte entre os achados e pesquisas compatíveis - com delineamento, amostragem e demais aspectos metodológicos comparáveis ao que foi realizado na pesquisa -, ou evidenciar as discrepâncias; caracterizar o estudo citado (delineamento, pessoa, tempo e lugar) de modo a esclarecer semelhanças e diferenças pertinentes a tal confrontação.

Ressalte-se a necessidade de discutir as possíveis explicações sobre os resultados com a literatura atualizada. A RESS sugere que sejam utilizados artigos científicos publicados nos últimos cinco anos.

Página 6, linha 37 - sexo é diferente de gênero. Observem que o estudo analisou a variável sexo. Revisar com atenção o texto.

O último parágrafo (curto e conciso) deve apresentar a conclusão da pesquisa com base do que se pode afirmar a partir dos resultados. Concluir com as implicações dos resultados do estudo para os serviços de saúde e/ou as políticas de saúde.

Página 6, linha 47 - está discutindo sobre a COVID-19, entretanto a Pesquisa Nacional de Saúde (2019) é anterior ao período pandêmico.

Página 6, linha 50 - evitem expressões genéricas (a literatura). Estabeleçam a conexão dos resultados com os artigos científicos, buscando semelhanças ou diferenças e explicações para os achados.

Página 6, linha 55 - poderiam explicar por que gênero foi considerado influência sociocultural. Acrescente-se que gênero não foi uma variável analisada no estudo.

Página 7, referências 17-19 - todas necessitam ser atualizadas. Uma das referências foi publicada há mais de 20 anos. Importante demonstrar atualização e conhecimento sobre as pesquisas atuais sobre o tema abordado no estudo.

Página 7, linha 11 - Quais são as evidências contrastantes? E o que isso quer dizer exatamente. Esse trecho poderia ficar mais compreensível.

Página 7, linha 12 - Quais são os indícios?

Página 7, linha 15 - quais são as tendências divergentes? Explicar e citas as referências que fundamentam as afirmativas. Por favor, informem quais são as outras pesquisas.

Página 7 - a vinculação do resultado com a literatura não ficou compreensível.

Página 7, terceiro parágrafo - Quais são os estudos prévios? Citar as referências.

Página 7, linha 36 - citar quais são os estudos.

Falta uma conclusão para o estudo. Um parágrafo conciso, que responda diretamente ao objetivo.

Referências

Necessitam de atualização e adequação as normas da RESS. Existem referências incompletas e em desacordo com as normas.

Seguir consistentemente as regras do estilo Vancouver, disponível em: http://www.nlm.nih.gov/bsd/uniform_requirements.html. Recomenda-se fortemente o uso de gerenciador de referências bibliográficas, como EndNote, Mendeley e Zotero para citar as referências no texto e automaticamente gerar a lista de referências nesta seção.

O formato para citar artigos científicos segue o padrão: Autor(es). Título. Nome abreviado do periódico. Ano;Volume(Número):Páginas inicial-final. (com a página final abreviada [ex.: 123-5]).

Todos os relatórios e literatura cinzenta devem ser rastreáveis, a fim de garantir transparência/acessibilidade. Incluir link de acesso, data de citação. Ex: Foley KM, Gelband H, editors. Improving palliative care for cancer [Internet]. Washington: National Academy Press; 2001 [cited 2002 Jul 9]. Available from: https://www.nap.edu/catalog/10149/improving-palliative-care-for-cancer

Tabelas e Figuras

Nas distribuições proporcionais, a soma das frequências nas categorias deve somar exatamente 100%. Observa-se na Tabela 1 variáveis, com total de 99% e outra com 100,1.

Verificar o somatório das frequências absolutas. Observou-se variáveis com totais inferiores 22.705. Se existem informações ignoradas na variável, precisa ser descrito.

Sobre a variabilidade descrita na página 5, linha 8, como foi mensurada?

O título da tabela 1, menciona características sociodemográficas, porém existem as comorbidades que estão descritas. Acrescente-se que a tabela desagrega os idosos em sem depressão e depressão, informação que precisa ser contemplada no título.

Incluir a fonte dos dados na tabela 1, figura 2, tabela 2.

A descrição da Tabela 2 no texto, apenas descreve o conteúdo. Faltam os dados numéricos.

Padronizar o número de casas decimais nos métodos, resultado e ilustrações: percentual 1 casa decimal, medida de associação: 2 casas decimais, p-valor: 3 casas decimais; apresentar p-valor exato com 3 casas decimais; ocorrências “0,000” devem ser grafadas como “<0,001”.

O conteúdo da Figura 3 - precisa descrito didaticamente no texto. Somente citar que um diagrama com os caminhos do modelo, não é suficiente.

Figura 3 - apresentar o significado de DAC e AVC.

Comentários menores

A redação do manuscrito deve ser feita sempre com o verbo no impessoal. Observou-se várias passagens que foi utilizado a primeira pessoa do plural.

A referência 1 pode ser atualizada. A RESS sugere-se que sejam utilizados artigos científicos publicados nos últimos cinco anos.

Página 2, linha 6 - necessita de referências.

Página 2, linha 10-13 - necessita de referências.

Página 2, linha 14 - informar a população de 2010, para que que todas as informações necessárias para o cálculo de variação estejam presentes.

A referência 4 (institucional) pode ser perfeitamente substituída por artigo científico.

Página 2, linha 15 - essa distribuição por sexo é realmente necessária? Em caso afirmativo, manter somente a maior população.

Página 2, linha 17 - substituir o termo “crucial” (característico de IA) por relevante.

Página 2, linhas 17-20 - necessita da referência que fundamente a afirmativa. Quais são os desafios específicos? Exemplificar.

Página 2, linha 22-24 - Incluir a referência que fundamenta a afirmativa.

Página 2, linha 24-25 - Incluir a referência que fundamenta a afirmativa.

A referência 5 (institucional) pode ser substituída por artigo científico.

A referência 6 - pode ser substituída por outra mais recente?

Página 2, linhas 33-34 - necessita de referências.

Página 2 - referência 7 - trata-se de um estudo realizado em Ghana, os seus resultados não devem ser generalizados. Para tanto, sugere-se citar um estudo de revisão sistemática.

Página 2, referência 6 - necessita de atualização. Trata-se de uma afirmativa importante que merece trazer estudos recentes.

Melhorar a conexão entre o terceiro e o quarto parágrafo da página 2.

Página 2, linha 42 - afirma-se estudos (plural), porém somente um foi citado.

Página 2, linha 44 - necessita das referências que fundamentam as afirmativas.

Página 2, linha 44 - o termo “curiosamente” não parece muito cinético. Acrescente-se que não ficou evidente o motivo da curiosidade. Seria interessante deixar esse trecho mais entendível.

Página 3 - Incluir a referência da Pesquisa Nacional de Saúde no item contexto.

Página 4, linha 39 e 30 - Incluir referência para o Critério de Informação Akaike e o Critério de Informação Bayesiano.

Substituir software por programa.

Página 5, substituir “incidência” por “frequência”.

Referência 11 - necessita de atualização.

Página 6, linhas 23-24, necessita de referências.

Página 6, linhas 40-43, necessita de referências.

Referência 14 - necessita de atualização.

Página 6, linha 54 - quais autores? Nenhum foi citado.

Referência 16 - necessita de atualização.

Referência 21 - necessita de atualização.

Referência 22 - necessita de atualização.

Referência 25 - necessita de atualização.

Recommendation: Major revision 

## Second round comments

O Núcleo Editorial da revista Epidemiologia e Serviços de Saúde (RESS) informa que a revisão do manuscrito intitulado “Dimensão latente de comorbidades e depressão em idosos: estudo transversal com dados da Pesquisa Nacional de Saúde, Brasil, 2019” (RESS-2025-0722.R1) foi concluída. O manuscrito foi considerado com potencial para publicação, mas reformulações maiores são necessárias. Solicitamos que apreciem a avaliação e realizem a revisão do manuscrito.

Para fazer a revisão do manuscrito, efetuem login no sistema e acesse o Author Center, onde o encontrará na lista “Manuscripts with Decisions”. Abaixo da barra “Actions”, clique em “Create a Revision”.

Lembrem-se de iluminar as mudanças realizadas no documento colorindo o texto manualmente com o amarelo. Uma vez terminada a revisão, o manuscrito deve ser submetido por meio do Author’s Center.

Todos os comentários do Núcleo Editorial e dos(as) revisores(as) devem ser considerados e respondidos, item por item, em carta aos editores. Para agilizar o processamento do manuscrito revisado, pedimos os comentários sejam bem específicos (informando página e linha). As sugestões que não forem consideradas deverão ser justificadas com fundamentação científica.

Solicita-se, gentilmente, que não deixem para enviar o manuscrito no último dia, pois o sistema não é nacional e sempre irá expirar o prazo no dia limite.

Agradecemos novamente pela submissão do artigo à RESS.

Cordialmente,

Núcleo Editorial

Epidemiologia e Serviços de Saúde: revista do Sistema Único de Saúde do Brasil

Site: www.scielo.br/ress

Comentários principais

A necessidade de revisão linguística persiste. Sugere-se que seja realizada por um(a) profissional e não pelo uso de IA. O objetivo necessita de clareza e harmonização com o título. Frases extensas, pouco coesas e que comprometem a clareza ainda foram identificadas. Existem resultados descritos no texto que não estão nas tabelas.

Resumo

O objetivo e o título devem guardar harmonia. Observa-se elementos (constructo novo e significativo, estado de saúde basal) que não aparecem no título. Acrescente-se que a parte final (após a vírgula) pode ser uma justificativa, no entanto não faz parte do objetivo da investigação. Nos resultados, 60 anos ou mais, consiste na população do estudo. Logo deve ser informada na seção de métodos. Os dados numéricos apresentados no resumo diferem daqueles relatados na seção de resultados. No texto as frequências estão descritas com duas casas decimais. No resumo com uma casa decimal. Deve-se padronizar o número de casas decimais nos métodos, resultado e ilustrações: percentual 1 casa decimal, medida de associação: 2 casas decimais, p-valor: 3 casas decimais; apresentar p-valor exato com 3 casas decimais; ocorrências “0,000” devem ser grafadas como “<0,001”.

A frequência absoluta deve anteceder a relativa.

A RP 1,27 não foi localizada no texto ou nas tabelas. Igualmente a RP do sexo feminino de 2,05 e a renda de 1,31.

Introdução

A informação sobre o sexo feminino e a depressão se repete na seção.

Necessário melhorar a conexão do parágrafo que trata do Modelo de Equações Estruturais Generalizadas com o anterior. O parágrafo que trata do modelo das equações, precisa de uma revisão, que demonstre com exatidão o que são e em que consiste a sua aplicação, contribuições. O parágrafo está genérico e bastante impreciso.

A hipótese não está compreensível. Não observável, não mensurável, pouco explorada. Como será validada ou refutada essa hipótese? Por favor, revisem.

A justificativa precisa anteceder o objetivo e informar precisamente qual a contribuição do estudo. Qual a sua relevância e inovação?

Métodos

Recomenda-se aderir consistentemente ao checklist STROBE para relato de estudos observacionais. A seção de métodos deve ser ajustada e as informações devem ser desmembradas e completadas de acordo com os seguintes itens: Delineamento do estudo; Contexto; Participantes; Variáveis (inserir todas as variáveis e categorias investigadas de forma idêntica ao que consta nos resultados e ilustrações); Fontes de dados/ mensuração; Viés; Tamanho do estudo; Métodos estatísticos. Acesse: https://www.strobe-statement.org/

Incluir referências que contemplem o plano amostral da pesquisa.

Todas as variáveis contidas nos resultados devem ser descritas na seção de métodos. Deve existir exata correspondência entre métodos e resultados.

Na análise estatística incluir a distribuição de frequências.

Resultados

Considerar a observação feita no resumo sobre o número de casas decimais.

As frequências absolutas devem anteceder as relativas no texto.

Na tabela 1, consta a prevalência de 11,83%, no entanto não consta o IC9%. Todos os dados citados no texto, devem estar contidos nas ilustrações (tabelas ou figuras).

Cada tabela deve ser descrita em um único parágrafo no texto.

As variáveis renda e co-residentes não foram descritas no texto.

O segundo parágrafo precisa chamar a Tabela.

A seção de resultados pode ser reduzida.

Referências

Sugere-se revisar e remover artigos publicados em revistas predatórias.

O número máximo de referências permitido pela RESS é 30. Por favor, revisem e retirem as referências excedentes.

Comentários menores

Página 2, linha 2 - retirar o vocábulo grande. Na redação científica deve-se evitar a utilização de adjetivos.

Página 2, linha 3 - remover “de forma expressiva”.

Página 2, linha 4 - colocar um ponto após idosas e incluir a referência que fundamenta a afirmativa.

Página 2, linha 6 - incluir comuns após mentais.

Página 2, linha 7 - remover o termo expressivo.

Página 2, linha 8 - a estimativa é 2020, não é recente. A última não faz sentido, uma vez que o parágrafo inicia citando estimativas no Brasil.

Página 2, linha 13 - Incluir as referências que fundamentam a afirmativa.

Página 2, linha 15 - substituir a referência institucional por artigo científico.

Página 2, linha 17 - Incluir um ponto final após (7).

Página 2, linha 17-21 - frase extensa, com muitas informações.

Página 2- primeiro e terceiro parágrafos estão iniciando da mesma forma.

Página 2, linha 24 - Incluir referência que fundamente a afirmativa.

Página 2, linha 31- Incluir referência que fundamente a afirmativa.

Página 2, linha 37- Incluir referência que fundamente a afirmativa.

Página 2, linha 39 - qual condição?

Página 2, linha 41 - quais padrões epidemiológicos estão se referindo?

Página 2, linha 41 - quais são os estudos? Informar detalhes dos estudos e incluir referências.

Página 3, linha 2 - quais são os estudos? Incluir as referências.

Página 3, linha 9 - remover o termo valiosa.

Página 3, linha 11 - a redação deve ser feita com o impessoal.

Página 3, linha 13 - quais associações? Quais variáveis? Quais características?

Página 3, linha 16 - qual contexto? Sugere-se remover a expressão “diante desse contexto”.

Página 6, linha 42 - remover o termo grande.

Página 8, linha 2 - remover “nossos”.

Página 8, linha 29 - remover “em síntese”.

Recommendation: Minor revision 

## Third round comments

O Núcleo Editorial da revista Epidemiologia e Serviços de Saúde (RESS) informa que a revisão do manuscrito intitulado “Dimensão latente de comorbidades e depressão em idosos: estudo transversal com dados da Pesquisa Nacional de Saúde, Brasil, 20199” (RESS-2025-0722.R2) foi concluída. O manuscrito foi considerado com potencial para publicação, mas reformulações menores são necessárias. Solicitamos que apreciem a avaliação e realizem a revisão do manuscrito. Para fazer a revisão do manuscrito, efetuem login no sistema e acesse o Author Center, onde o encontrará na lista “Manuscripts with Decisions”. Abaixo da barra “Actions”, clique em “Create a Revision”. Lembrem-se de iluminar as mudanças realizadas no documento colorindo o texto manualmente com o amarelo. Uma vez terminada a revisão, o manuscrito deve ser submetido por meio do Author’s Center. Todos os comentários do Núcleo Editorial devem ser considerados e respondidos, item por item, em carta aos editores. Para agilizar o processamento do manuscrito revisado, pedimos os comentários sejam bem específicos (informando página e linha). As sugestões que não forem consideradas deverão ser justificadas com fundamentação científica.

Solicita-se, gentilmente, que não deixem para enviar o manuscrito no último dia, pois o sistema não é nacional e sempre irá expirar o prazo no dia limite.

Agradecemos novamente pela submissão do artigo à RESS.

Cordialmente,

Núcleo Editorial

Epidemiologia e Serviços de Saúde: revista do Sistema Único de Saúde do Brasil

Site: www.scielo.br/ress

Comentários principais

Resumo

Objetivo

O trecho sobre a Pesquisa Nacional de Saúde consiste em uma informação metodológica, logo deve ser relatado na seção seguinte.

Métodos

Sugere-se “Estudo transversal com dados da Pesquisa Nacional de Saúde no Brasil de 2019 ...

Resultados

Os resultados devem trazer os principais achados da investigação. Nesse sentido, observou-se que existem dados que estão citados no resumo em constam na tabela suplementar. A sugestão é que a tabela suplementar seja integrante do corpo do manuscrito e outra ilustração seja exposta como suplementar.

Aspectos éticos

Citar também a Resolução 466/2012. Sugere-se excluir a referência 1, não é necessária.

Introdução

A primeira referência citada na seção deve ser a número 1. Revisar toda a numeração. A introdução faz uma mescla de informações que são gerais ou de estudos mais globais com os específicos do Brasil, sem realizar a distinção entre ambos. Revisar a seção para evidenciar as especificidades. No objetivo está mantida a sugestão feita no resumo.

Métodos

Recomenda-se aderir consistentemente ao checklist STROBE para relato de estudos observacionais. A seção de métodos deve ser ajustada e as informações devem ser desmembradas e completadas de acordo com os seguintes itens: Delineamento do estudo; Contexto; Participantes; Variáveis (inserir todas as variáveis e categorias investigadas de forma idêntica ao que consta nos resultados e ilustrações); Fontes de dados/ mensuração; Viés; Tamanho do estudo; Métodos estatísticos. Acesse: https://www.strobe-statement.org/

No item contexto incluir as referências sobre a Pesquisa Nacional de Saúde.

A construção da variável “comorbidades” não ficou suficientemente compreensível. Descrevem com exatidão e detalhes a sua elaboração. Essa variável é essencial para toda a análise.

Resultados

Persiste a orientação para a padronização das casas decimais. Na tabela 1, os percentuais estão com duas casas decimais. Padronizar o número de casas decimais nos métodos, resultado e ilustrações: percentual 1 casa decimal, medida de associação: 2 casas decimais, p-valor: 3 casas decimais; apresentar p-valor exato com 3 casas decimais; ocorrências “0,000” devem ser grafadas como “<0,001”.

Verificar os totais % das variáveis da tabela1. Em uma delas o total está 99,99%.

Na tabela 1, algumas variáveis apresentam informação ignoradas. Sugere-se que essas o número e o% de ignorados dessas variáveis sejam apresentados em nota abaixo da tabela.

Persiste a divergência entre os dados exposto na Tabela 1 e a sua descrição no texto. No corpo da tabela os % apresentam duas casas decimais. A RESS recomenda uma casa decimal, como citado no parecer anterior. No texto os % estão relatados com uma casa decimal.

Cada tabela deve ser descrita em um único parágrafo no texto. Essa recomendação já existia na revisão anterior.

Parágrafo 3 - não cita a ilustração na qual se encontram os resultados.

Existem parágrafos na seção que não mencionam a ilustração na qual se encontram os dados. Por favor, revisem com atenção esse ponto.

A tabela suplementar 1, deve ser integrada ao texto. Os(as) autores(as) devem identificar uma outra ilustração que possa ser exposta como material suplementar. Os resultados dessa tabela são relevante e foram selecionados pelos(as) próprios(as) autores(as) para integrar o resumo do manuscrito. Página 6, linhas 9 e 10 - a descrição do resultado sobre a idade não ficou compreensível. Acrescente-se que o resultado está repetido no texto. Os resultados da idade do sexo masculino e do feminino são exatamente os mesmos? Por favor, verifiquem esse resultado na tabela. Página 6, linhas 24-26 - A frase que inicia com “um escore... é uma interpretação do resultado. A variável escore latente não deveria ser chamada de comorbidades nas ilustrações e no texto?

Discussão

O segundo parágrafo da seção deve apresentar as limitações do estudo.

Página 6, segundo parágrafo -incluir as referências que fundamentam as afirmativas feitas no texto.

As limitações do estudo devem contemplar o uso de dados secundários. E atualidade dos dados. Estamos em 2026.

Comentários menores

Página 2, linha 4 - incluir o ano da estimativa.

Página 2, linha 6 - Por favor, revisem a afirmativa que se trata de um dos transtornos mentais mais comuns. Depressão e ansiedade fazem parte dos transtornos mentais comuns. Talvez trata-se de um dos transtornos mentais comuns mais recorrentes ou Trata-se de um dos transtornos mentais mais recorrentes.

A referência 6 não trata sobre o Brasil.

Página 2, linha 8 - a referência 4, trata especificamente do Brasil e a frase é geral. Talvez manter apenas a 5 aqui e deslocar a 4 para a frase que trata do país.

Página 2, linha10 - remover a informação sobre países de baixa e média renda, uma vez que desejam apresentar a informação do Brasil.

Página 2, linhas 11-13 - incluir a referência que fundamenta a afirmativa.

Página 2, linhas 13-18 - frase extensa.

Página 2, linhas 13 e 18 - iniciam da mesma forma.

Página 2, linha 21 - a referência 8 não trata do Brasil e o parágrafo se refere ao país.

Página 2, linha 25 - incluir a referência que fundamenta a afirmativa.

Página 2, linha 34 - essa afirmativa se refere aos estudos realizado no Brasil, referências 4 e 15 e isso necessita ficar explicito no texto.

Página 2, linha 40 em diante - frase demasiadamente extensa.

Página 2, linha 40 - modificar o início da frase, precisa informar que no Brasil.

Página 3, linha 1 - a referência 15 aqui, deveria ser substituída por uma que abordasse especificamente os resultados da Pesquisa Nacional de Saúde.

Página 3, linha 1 - Quem identificou?

Página 3, linha 3 - incluir a referência que fundamenta a afirmativa.

Página 3 - O parágrafo da justificativa e o do objetivo podem formar um único.

Página 3, linha 15 - Remover “nesse contexto” e presente estudo. Sugestão: o estudo teve por objetivo...

Página 3, linha 23 - remover “mais recente”, informar o ano de 2019 é suficiente.

Página 7, linha 2 - remover o termo corroborar. É muito forte tem o sentido de comprovar.

Página 7, linha 12 - evitar expressões genéricas (a literatura). Idem linha 23.

Página 7, linhas 16 e 35 - iniciam da mesma forma.

Página 8, linha 1 - retirar o termo grande.

Recommendation: Minor revision 

